# Thermal Characterisation and Toxicity Profile of Potential Drugs from a Class of Disubstituted Heterofused Triazinones

**DOI:** 10.3390/molecules30030506

**Published:** 2025-01-23

**Authors:** Małgorzata Sztanke, Renata Łyszczek, Agnieszka Ostasz, Halina Głuchowska, Krzysztof Sztanke

**Affiliations:** 1 Department of Medical Chemistry, Medical University of Lublin, 4A Chodźki Street, 20-093 Lublin, Poland; malgorzata.sztanke@umlub.pl; 2 Department of General and Coordination Chemistry and Crystallography, Institute of Chemical Sciences, Faculty of Chemistry, Maria Curie-Skłodowska University in Lublin, M.C. Skłodowskiej Sq. 2, 20-031 Lublin, Poland; renata.lyszczek@mail.umcs.pl (R.Ł.); agnieszka.ostasz@mail.umcs.pl (A.O.); halina.gluchowska@mail.umcs.pl (H.G.); 3 Laboratory of Bioorganic Compounds Synthesis and Analysis, Medical University of Lublin, 4A Chodźki Street, 20-093 Lublin, Poland

**Keywords:** disubstituted heterofused triazinones, potential analgesics, potential anticancer agents, dual-acting agents, thermal characterisation, thermal degradation mode, TG/DSC, TG/FTIR, in vivo and ex vivo toxicity profile, antihaemolytic activity

## Abstract

The thermal characterisation and toxicity profile of a class of disubstituted heterofused triazinones were revealed in this article for the first time. The thermal behaviour of molecules **1**–**12** was investigated by means of TG and DSC analyses performed in an air atmosphere and by the coupled TG/FTIR technique in a nitrogen atmosphere. The heating atmosphere affects both the stability of compounds and the degradation mechanism. A two-step degradation occurs in air, while a one-step degradation takes place in nitrogen, both preceded by a melting process. Compound **3** shows the highest thermal stability, while molecule **10**—the lowest. The thermal decomposition of the studied heterocyclic molecules begins with the degradation of the bicyclic system, resulting in the formation of volatile gaseous products such as ammonia/hydrazine, hydrogen cyanide, carbon dioxide, and isocyanates. In the further stage, mainly aromatic compounds are released, and their chemical composition depends on the presence and type of substituents at the phenyl and benzyl moieties. In addition, the toxicity profiles of molecules were assessed in the animal (zebrafish) and cellular (erythrocytes) models, and the antihaemolytic activity was evaluated in the AAPH- and H_2_O_2_-induced haemolysis inhibition assays. It was found that all the tested compounds are safe for the developing zebrafish and red blood cells, and they are able to effectively protect erythrocytes from oxidative damage. These favourable properties make them promising drug candidates suitable for further in vivo studies.

## 1. Introduction

**Figure 1:**
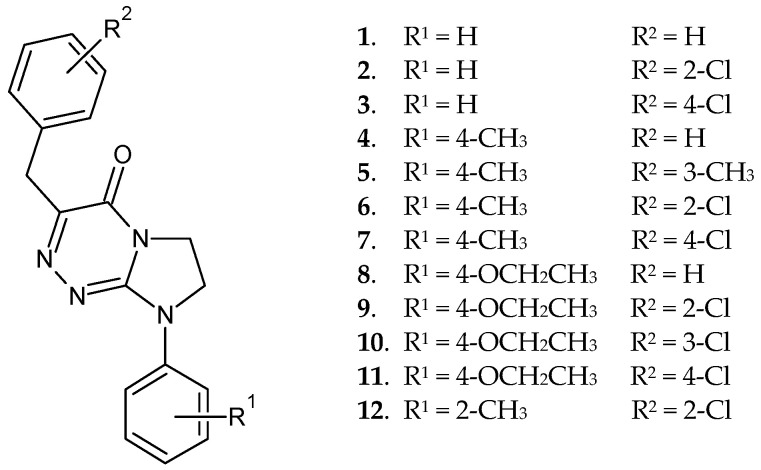
Structures of the studied compounds.

Disubstituted heterofused 1,2,4-triazinones (**1**–**12**) (Figure 1), belonging to an important class of potential drug molecules, have been shown to possess established structures in solution and solid state [1]. Compounds **1**, **2**, **6**, **8**, and **9** revealed both analgesic activities in the central nervous system (CNS) and were low in acute toxicity for mice. Molecules **2** and **8** (producing the most potent effects in reversing writhing episodes caused by intraperitoneal injection of acetic acid) were found to be the promising potential analgesics [1]. Additionally, heterocycles **6** and **9** were identified as dual-acting agents. Beside their mentioned antinociceptive activities in vivo, they also demonstrated antiproliferative effects in human tumour cells of the breast, similarly as molecule **11** [2]. In previous and recent biomimetic chromatographic studies, we have proved—on the basis of highly predictive QSAR equations—that all the presented compounds (**1**–**12**) are able to easily penetrate cell membranes (including the blood-brain barrier) due to their optimal lipophilicity, polarity, and molecular size [3,4]. In addition, in behavioural tests we have demonstrated the ability of these less polar, more lipophilic, and bigger-size molecules to cross the blood-brain barrier through their in vivo antinociceptive effects on the CNS in mice [1]. These favourable biopharmaceutical properties have made the title molecules promising potential analgesics and anticancer agent candidates worthy of further investigation in the preclinical phase of drug development. Bioavailability is of increasing concern to medicinal chemists, as it increases the potency and efficacy of the potential drug.

Some thermal studies carried out on pharmacologically or energetically important heterofused 1,2,4-triazines (as-triazines) have been described in the literature. The topics of the published papers were focused on investigating their thermal properties and the mode of decomposition upon controlled heating [5,6,7,8,9,10,11,12]. In addition, our previous thermal analyses have proved that heterofused structures incorporating the completely conjugated as-triazinone (1,2,4-triazinone) template with a bridgehead nitrogen atom, are of high purity, do not undergo polymorphic transformations in the solid state, and are stable at temperatures much higher than ambient temperature [5]. The above results prompted us to focus our attention and extend our thermal investigations to another class of polyazaheterocycles having the fully conjugated as-triazinone core incorporated in their bicyclic structure, and thus presumably stable molecules. Our research team is constantly looking for pharmacologically active molecules with high thermostability, as this thermal feature may be desirable during their storage and further processing as potential drugs. In addition, based on the decomposition kinetics of three approved drugs (i.e., acyclovir, azidothymidine, and amiodarone hydrochloride) that were stable at temperatures significantly above ambient temperature, it was proven that storage at 20–45 °C in air under normal atmospheric pressure did not practically shorten their shelf life [13,14]. Taking the above into account, it can be assumed that both these drugs and innovative molecules containing the completely conjugated as-triazinone template should be stable in the solid state even under more adverse conditions in a variety of climatic zones.

There is still a research gap in the thermal characterisation of imidazolidinoannelated molecules with a fully conjugated as-triazinone system incorporated in their bicyclic structure. None of the papers to date has focused on thermal studies of analgesic and/or anticancer active drug candidates **1**–**12**. Although we have investigated these compounds for their possible pharmaceutical use [1,2,3,4], their thermal analyses have not been performed and reported to date. Therefore, nothing is known about their thermal behaviour and degradation mode upon heating. Undoubtedly, knowledge about their thermostability and key thermal properties will be an important element of their further characterisation in the preclinical phase of drug development. Thus, thermal studies of these molecules are absolutely justified.

As thermal analysis methods are essential in the thermal characterisation of molecular pharmaceuticals, drug candidates, potential drugs, and phase change materials [15,16,17,18,19,20,21], we have applied thermogravimetry/differential scanning calorimetry as well as thermogravimetry coupled with Fourier transform infrared spectroscopy in the present studies. The rationale for choosing these combined and coupled thermal analysis techniques is the fact that they enable the credible interpretation of each phenomenon occurring with mass loss and energy changes while heating the test sample at a constant rate, the control of its purity and understanding the mode of its decomposition [15]. Using these techniques, important characteristics of the molecules such as stability, solubility, polymorphism, and interaction with excipients can be investigated [15,22,23,24,25,26].

The novelty of the current paper is the detailed thermal characterisation of 12 disubstituted heterofused triazinones with prospective medical use. Thermal studies on this class of molecules have been performed for the first time. In this article, we document the thermal behaviour and properties of the investigated compounds in oxidative conditions (i.e., those that prevail in air), and also explain their thermal degradation mode, identifying the volatile decomposition products in inert conditions (i.e., in nitrogen). Understanding the thermal behaviour of these potential drugs will be of great practical and scientific importance. The study results will be of practical usefulness in the case of the pharmaceutical approval of disubstituted heterofused triazinones. They will make it possible to establish the optimal storage conditions for compounds in this class. In addition, they will allow the determination of the optimal thermal conditions for the controlled thermal utilisation process of each molecule that the emitted volatile degradation products do not cause air pollution and consequently do not have an unfavourable impact on the environment. On the other hand, the results presented in this article will be a significant contribution to expanding the current knowledge about the thermal properties and the decomposition mode of heterobicycles with an incorporated conjugated as-triazinone system.

Moreover, compounds with potential therapeutic use, in order to be further developed as drug candidates, must be thoroughly tested—in the preclinical phase of drug development—for their safety and toxicity. For this purpose, it is necessary to conduct multidirectional toxicological studies on various experimental models—both cellular and animal. Due to the fact that so far only acute toxicity in mice of some disubstituted heterofused triazinones has been determined, an assessment of the toxicity profile of this class of compounds is fully justified. The novelty of the biological part of this paper is also the toxicological research carried out on the animal (early-life stages of zebrafish (*Danio rerio*)) and cellular (isolated red blood cells) models, which should allow for the selection of the most promising compounds for further, more advanced in vivo studies. This will increase the possibility of their potential therapeutic use. The rationale for conducting the in vivo and ex vivo investigations is the fact that the described structurally related annelated triazinones were low in toxicity for zebrafish and erythrocytes and were able to inhibit haemolysis caused by the action of reactive oxygen species [27].

Based on the results of multidirectional thermal and toxicological studies, it will be possible to decide whether the title disubstituted heterofused triazinones are suitable for further development in the drug research process.

## 2. Results and Discussion

### 2.1. TG/DSC Analysis of Disubstituted Heterofused Triazinones (***1**–**12***) in Air

The thermal analysis in air was carried out using techniques such as differential scanning calorimetry (DSC) and thermogravimetric analysis (TGA), which provide valuable insights into the compound’s stability and purity.

**Figure 2:**
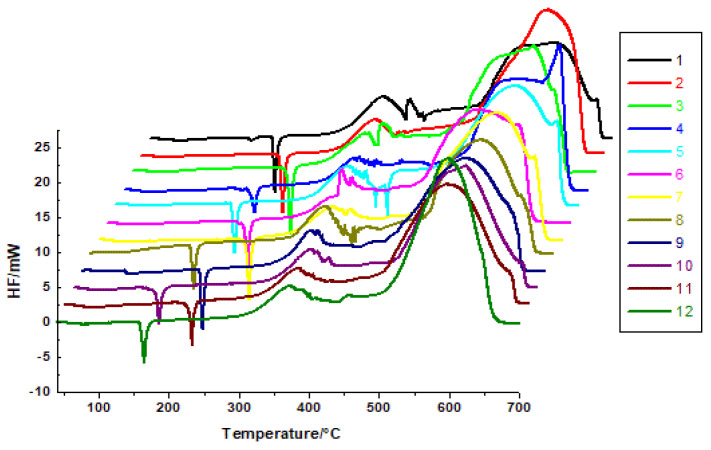
DSC curves of disubstituted heterofused triazinones (**1**–**12**).

The melting point of a compound (i.e., the temperature at which its solid and liquid phase are in equilibrium) is a critical property for crystallisation, formulation development, and quality control. The peak observed in the heat flow curve corresponds to the melting point (T_peak_) of the tested compound (Table 1). The sharpness of the DSC peak in the case of disubstituted heterofused triazinones indicates their purity. The parent structure **1**, which contains no substituents (R^1^ = H, R^2^ = H) attached to the phenyl and benzyl moiety, has a T_peak_ of 219 °C. Replacing the hydrogen atom at the benzyl moiety with a chlorine atom (compound **3**), and introducing substituents in the *para* positions—a methyl group to the phenyl ring and a chlorine atom to the benzyl formation (compound **7**)—significantly shifts the melting points to higher temperatures (Table 1, Figure 2). These structural modifications in the case of compounds **3** and **7** may likely lead to a stronger intermolecular interaction and/or more efficient packing in the crystal lattice, thus resulting in higher melting points. The combination of these electron-withdrawing and electron-donating groups causes more structured and stable packing, contributing to the observed increase in melting points. In turn, the melting points below 200 °C are observed for the investigated structures **8**, **10**, and **12**. In the case of these compounds, the *meta*-chloro and *ortho*-chloro substitution on the benzyl formation (structures **10** and **12**, respectively), as well as the *para*-ethoxy (molecules **8** and **10**), and *ortho*-methyl substitution (compound **12**) at the phenyl ring, are observed. However, the T_peaks_ of the remaining derivatives (**1**, **2**, **4**, **5**, **6**, **9**, **11**) range from 209 to 242 °C.

The melting enthalpy (ΔH_m_) values obtained under oxidative conditions range from 21.08 kJ·mol^−1^ to 52.82 kJ·mol^−1^ (Table 1). The highest ΔH_m_ values were observed for compounds **9** and **3**. It is seen that derivative **9** contains an ethoxy group in *para* position of the phenyl ring and a chlorine atom in *ortho* position of the benzyl moiety, while congener **3** has an unsubstituted phenyl ring and a *para*-chloro group on the benzyl moiety. It is worth emphasizing that molecule **3** exhibits the highest melting point among all 12 disubstituted heterofused triazinone derivatives.

**Figure 3:**
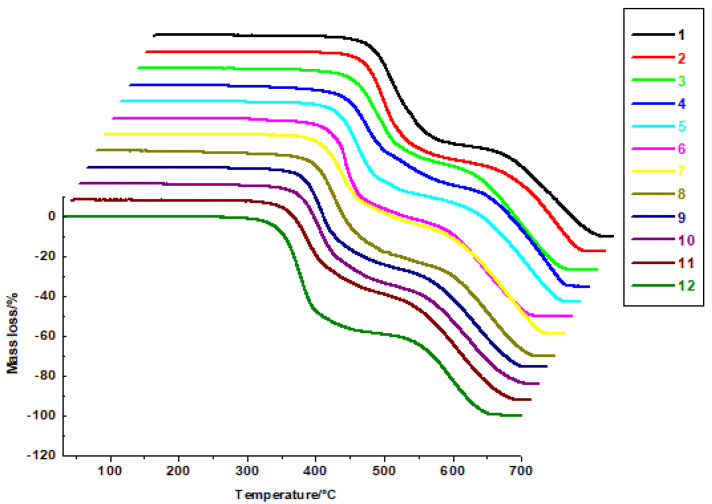
TG curves of disubstituted heterofused triazinones (**1**–**12**).

The TG curves (Figure 3) show two main stages of decomposition. The first mass loss, observed for all disubstituted heterofused triazinones, was associated with the formation of unstable decomposition products. At this stage, the most stable were molecules **2** and **3**, whose decomposition occurred at temperatures above 275 °C and 276 °C, respectively. Additionally, a smaller exothermic peak is observed on the DSC curve. The final stage of degradation occurs above 453 °C for compounds **3** and **9**, which show the lowest decomposition onset temperature compared to the remaining molecules. During this stage, the decomposition process is associated with a large exothermic peak on the DSC curve. The final decomposition temperature, where a plateau is observed on the TG curve, ranges from 641 °C to 690 °C (Table 2, Figure 3).

The thermal analysis results in oxidising conditions proved that disubstituted heterofused triazinones are pure molecules with high thermostability. This means that their storage will not require any special recommendations. Moreover, based on literature data on thermally stable drugs [13,14], it can be assumed that the storage of the investigated compounds at temperatures in the range of 20–40 °C should not practically affect their shelf life. This makes them suitable for possible future pharmaceutical applications. To repay the significant costs on research and development, each drug candidate must be acceptable even in more unfavourable conditions of climate zones. Therefore, this promising class of potential drugs with beneficial thermal properties appears to be suitable for further research in the drug development process.

### 2.2. TG/FTIR Analysis of Disubstituted Heterofused Triazinones (***1**–**12***) in Nitrogen

**Figure 4:**
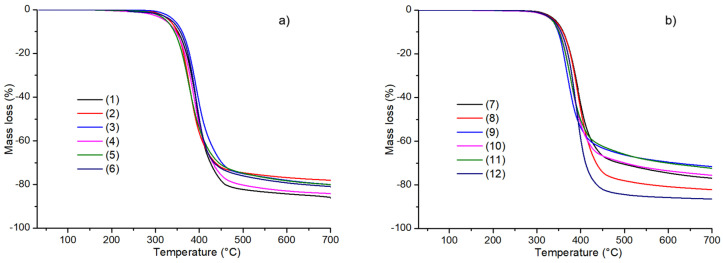
TG curves of compounds: (**a**) **1**–**6**, (**b**) **7**–**12** recorded in air.

The thermogravimetry (TG) coupled with Fourier transform infrared spectroscopy (FTIR) were applied for determination of the thermal behaviour of compounds **1**–**12** during their heating in nitrogen. The TG analysis (Figure 4) enabled the assessment of their thermal stability in an inert atmosphere, which varied in the following order: **10** (156 °C) < **4** (163 °C) = **5** (163 °C) < **9** (173 °C) < **1** (194 °C) < **8** (204 °C) < **12** (206 °C) ≅ **11** (207 °C) < **2** (212 °C) < **6** (228 °C) < **7** (249 °C) < **3** (255 °C). Considering the above data, several conclusions can be drawn regarding the influence of the attached substituents (R^1^ and R^2^) at the phenyl and benzyl moieties on the thermal stability of particular molecules. In the case of derivatives **1**–**7** and **12**, containing H, 4-CH_3_, 2-CH_3_ as R^1^ and H, 2-Cl, 4-Cl, 3-CH_3_ as R^2^, the introduction of *para*- or *ortho*-chloro groups (as R^2^) has a beneficial effect on their stability. These chlorine substituents enhance the thermostability in relation to the parent structure **1**. The introduction of one or two methyl substituents into the structure of heterofused triazinones leads to a decrease in their thermal stability compared to the unsubstituted compound **1**. Molecules **3** and **7** exhibit the highest thermal stability, which is associated with the presence of H or 4-CH_3_ (as R^1^) and 4-Cl (as R^2^). In the case of compounds **8**–**11** bearing a *para*-ethoxy group at the phenyl moiety, their thermostability is strongly affected by R^2^ such as H, 2-Cl, 3-Cl, or 4-Cl. Heterocycles **8** and **11** (with H or 4-Cl as R^2^, respectively) show the higher thermal stability in comparison to the parent structure. On the other hand, in the case of molecules substituted with the ethoxy group as R^1^, the introduction of a chloro substituent at positions 2 or 3 of the benzyl moiety (structures **9** and **10**) leads to a significant decrease in their thermal stability compared to **1**. The heterofused triazinone **10** shows the lowest thermal stability due to specific structural features connected with the presence of *para*-ethoxy and *meta*-chloro group at the phenyl and benzyl moiety, respectively. The compound **3**—with the unsubstituted phenyl ring and the *para*-chloro-substituted benzyl moiety—displays the highest thermal stability. The data obtained from the thermal analysis in nitrogen indicate that, in the case of molecules under consideration, the impact of the presence and type of substituents on the stability of compounds is very significant. It seems that the effect of these substituents is more related to steric factors than to their nature.

Further heating of disubstituted heterofused triazinones **1**–**12** in nitrogen led to the decomposition process with a significant mass loss in the range of 72.8–86.6%, which took place up to about 500 °C. At higher temperature, for all the compounds only small mass losses in the range of 2.2–6.2% were observed. Owing to the non-oxidising heating atmosphere, solid residues such as carbon and some organic compounds were formed. The masses of the solid residues change in the following order: **12** (13.4%) < **1** (14.0%) < **4** (15.8%) < **8** (17.5%) < **6** (19.0%) < **3** (19.9%) < **5** (20.0%) < **2** (21.9%) < **7** (22.6%) < **10** (23.9%) < **11** (27.2%) < **9** (27.9%). As can be seen from the above data, the largest masses of residues were observed for the investigated heterocycles with the highest molecular weight. In turn, the smallest solid residue was noticed for compound **12**, which is not characterised by the lowest molecular weight. This can be correlated with the different position (*ortho*) of the methyl group at the phenyl moiety that strongly influences the thermal degradation pathway.

**Figure 5:**
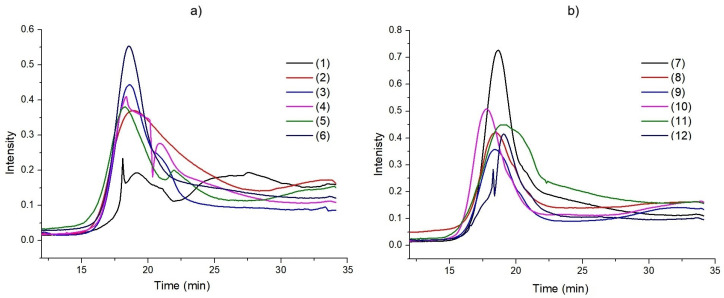
The Gram–Schmidt plots for compounds: (**a**) **1**–**6**, (**b**) **7**–**12**.

Simultaneously with the thermogravimetric analysis of heterofused triazinones in nitrogen, the infrared spectra of the released volatile decomposition products during heating were recorded. The Gram–Schmidt curves given in Figure 5 illustrate the relationship between the release intensity of volatile products and the time (temperature).

**Figure 6:**
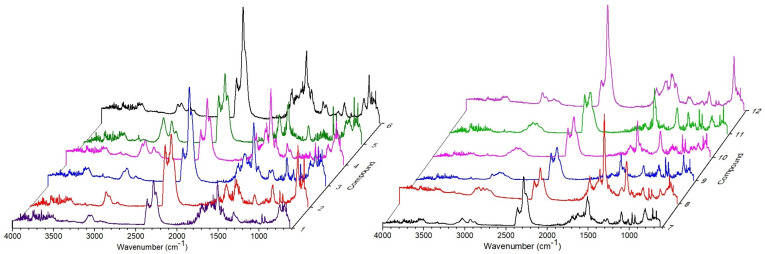
The FTIR spectra of volatile thermal decomposition products of compounds **1**–**12** in nitrogen recorded at 19 min of analyses.

The maximum intensity of gases release occurs within a time range of 17.8–19.2 min corresponding to the observed significant mass losses. The description of volatile decomposition products of the title heterocycles will be made in relation to the infrared spectra of all compounds recorded at the same time (19 min) in order to better notice the differences between them (Figure 6). We will start with a description of the volatile products of their decomposition from parent compound **1**. The first volatile degradation products of molecule **1** are the results of breakage of heterofused 1,2,4-triazinone template, yielding in the formation of compounds containing nitrogen atoms. The broad bands in the region of 3400–3200 cm^−1^ with maxima at 3330 and 3272 cm^−1^, as well as the weak bands in the ranges of 1500–1350 cm^−1^ and 800–650 cm^−1^ with a maximum at 713 cm^−1^, can be ascribed to the stretching vibrations of hydrogen cyanide [28,29]. The FTIR spectra also show the characteristic band at 3333 cm^−1^ and the double band at 965 and 930 cm^−1^ from the stretching and deformation vibrations of hydrazine and/or ammonia molecules. Other degradation products of the bicyclic skeleton are isocyanic acid and its derivatives, which give the bands in the ranges of 3650–3470, 2300–2100 (submaxima at 2284 and 2251 cm^−1^), and 850–600 cm^−1^ assigned to the stretching vibrations of NH groups, the stretching vibrations of NCO moieties, as well as the bending vibrations of HNC and NCO groups. Additionally, carbon dioxide molecules are released due to the diagnostic bands in the range of 2400–2300 cm^−1^ and at 668 cm^−1^ [30]. Decomposition of the heterocyclic scaffold also occurs with the release of some carbonyl-containing compounds, which is confirmed by the presence of a band at about 1700 cm^−1^. The bands at 3031, 2930, 2888 cm^−1^, and those at 1456 cm^−1^ corresponding well with the stretching vibrations of C–H groups in aromatic rings and the stretching and deformation vibrations of methyl group of toluene were identified [31]. The attendance of the stretching modes of aromatic C_Ar_C_Ar_ ring was confirmed by the bands at 1616 and 1506 cm^−1^. The in-plane and out-of-plane bending vibrations of CH groups of the monosubstituted benzene ring are observed at 1084, 1033, and 745 cm^−1^, respectively. In addition to the products mentioned above, primary and/or secondary aromatic and aliphatic amines are released as the volatile decomposition products of compound **1.** The FTIR spectra exhibit also the bands at 1307 and 1271 cm^−1^ from the stretching vibrations of C–N groups that are diagnostic for amine compounds. These bands can be ascribed to amines such as aniline, *N*-methylaniline, *N*,*N*-dimethylaniline, and trimethylamine [32]. Additionally, multi-peak bands in the wavenumber ranges of 4000–3500 cm^−1^ and 1800–1350 cm^−1^ appear due to the stretching and deformation vibrations of water molecules.

Considering the character of the evolved gaseous decomposition products of the remaining compounds, it can be postulated that the first stage of degradation is connected with the breaking of bonds in the heterofused triazinone template with the emission of gases such as NH_3_, HCN, N_2_H_4_, CO_2_, H_2_O, isocyanic acid, and its derivatives, as observed for **1**. Next, various aromatic degradation products of the studied compounds are released. Their chemical character is strictly connected with the kind and position of R^1^ and R^2^ substituents. In the description of the volatile thermal degradation products of compounds **2**–**12** we will focus on the identification of the emitted gases characteristic for each compound as well the diversity among them connected with the structural features of the compounds under investigations. The FTIR spectrum of volatile degradation products of heterocycle **2** is dominated by the bands at 3508, 3056, 3043, 2937, 1683, 1616, 1498, 1507, 1306, 1272, 1095, 1046, 1013, 806, and 727 cm^−1^ assigned to the stretching vibrations of NH, C_Ar_H, CH, and C_Ar_C_Ar_ groups as well as the deformation vibrations of CH_3_ and CN groups, out-of-plane CH vibrations of aromatic rings, as well as the stretching vibrations of C–Cl groups, respectively. These bands can be ascribed for aromatic species, especially amines and *ortho*-chlorotoluene [33]. The decomposition of molecule **3**, besides the emission of inorganic species, is accompanied by the release of aromatic compounds, especially aniline. The FTIR spectra of volatile degradation products of structure **4** at the highest intensity of their evolution are dominated by the bands at 3545, 3333, 3067, 3032, 2933, 2920, 2358, 2283, 2252, 1683, 1558, 1507, 1446, 1309, 1032, 965, 930, 807, 727, 713, 698, and 668 cm^−1^. Besides small molecules containing nitrogen atoms, the highest intensity is observed for bands connected with the stretching and deformation vibrations of amine, aromatic, and methyl moieties and most probably *para*-toluidine [34]. The infrared spectra of volatile degradation products of compound **5** exhibit very strong bands at 3030, 2930, 1616, 1521, 1350, as well as at 766 cm^−1^, which can be attributed to the emission of *meta*-xylene [35]. Besides *meta*-xylene, *para*-toluidine and/or aniline are also evolved. The FTIR spectrum of the emitted gases from heterocycle **6** shows bands connected with the release of HNCO and/or phenyl isocyanate due to the presence of bands at 3545, 2283, 1309, and 746 cm^−1^. It is worth mentioning that in comparison to the described above compounds **1**–**5**, the diagnostic bands derived from NH_3_/N_2_H_4_ are very weak. This observation may indicate a different mechanism of the thermal decomposition of structure **6**. The infrared spectrum exhibits also bands at 3548, 3072, 2935, 1684, 1558, 1507, 1446, 1271, 1122, 1048, and 811 cm^−1^ ascribed to the vibrations of *para*-toluidine (i.e., *para*-methylaniline) and *ortho*-chlorobenzene. The infrared spectrum of volatile decomposition products of molecule **7** is predominated by the bands derived from *para*-chlorotoluene and *para*-methylaniline. The most intense bands appear at 3031, 2930, 2285, 1507, 1304, 1270, 1095, 1012, 965, 930, 807, 713, and 668 cm^−1^.

The presence of the ethoxy group at the phenyl ring in molecules **8**–**11** enhances the emission of different volatile products of their decomposition. Their FTIR spectra are prevailed by the bands derived from the released *para*-phenetidine (i.e., *para*-ethoxyaniline) and its derivatives. The broad band in the wavenumber range of 3100–2750 cm^−1^ (submaxima at 3076, 2987, and 2939 cm^−1^), as well the bands at 1683, 1558, 1507, 1236, 1177, and 1033 cm^−1^, were assigned to the stretching vibrations both from the C–H groups of an aromatic ring and methyl and methylene groups, the stretching vibrations of C_Ar_C_Ar_ bonds, as well as the stretching vibrations of C–O–C and C–O groups. Among gaseous products of thermal decomposition of compounds **9**–**11,** chlorotoluene molecules are released. The thermal degradation of heterocycle **12** occurred with an intense emission of isocyanide compounds along with *ortho*-chlorotoluene, as can be seen from its FTIR spectrum.

The FTIR spectra of volatile products of compounds **3**, **6**, **7**, and **9** recorded above 500 °C allowed recognition of the bands of low intensity in the wavenumber range of 3050–2650 cm^−1^ due to the release of hydrochloric acid. Also at higher temperature, the emission of carbon monoxide molecules occurred during decomposition of heterocycles **5**, **10**, and **11**. The infrared spectra show the diagnostic bands at 2184 and 2107 cm^−1^ from carbon monoxide vibrations. The release of methane molecules was observed for compounds **5** and **6** due to the presence of characteristic bands with a maximum at 3015 cm^−1^ from the stretching vibrations of C–H groups of methane [36,37].

### 2.3. The Toxicity Profile of the Selected Compounds (***1***, ***2***, ***6***, ***8***, ***9***, ***11***, and ***12***) to the Developing Zebrafish

The tested potential analgesic and anticancer agents from the class of disubstituted heterofused 1,2,4-triazinones [1,2] are characterised by optimal lipophilicity, polarity, and molecular size, as well as biopharmaceutical properties important from the point of view of pharmacokinetics [3,4]. Therefore, it is reasonable to assess the safety/toxicity profile in various models (both animal and cellular) of these pharmacologically relevant compounds in the preclinical phase of drug development. Taking this into account, molecules with the highest activity and favourable properties were selected for the in vivo FET (Fish Embryo Toxicity) assay [38] in the zebrafish (*Danio rerio*) model, which is commonly used in toxicological studies in drug discovery and development [39,40,41,42,43,44]. To determine the acute toxicity of tested heterofused triazinones on developing zebrafish, lethal and sub-lethal endpoints were assessed. At the end of the exposure period (96 h post-fertilisation), mortality (as well as MNLC—the maximal non-lethal concentration and LC_50_—the half maximal lethal concentration), hatchability, heart rate, and developmental abnormalities in *Danio rerio* larvae were determined for molecules **1**, **2**, **6**, **8**, **9**, **11**, **12** (which may mimic innovative nucleobases of the antimetabolite-type) and the standard drug pemetrexed (a clinically approved anticancer agent belonging to the folate antimetabolites). Based on these endpoints, the no observed adverse effect concentration (NOAEC) and the lowest observed adverse effect concentration (LOAEC) values were established in compound/pemetrexed-treated groups.

**Figure 7:**
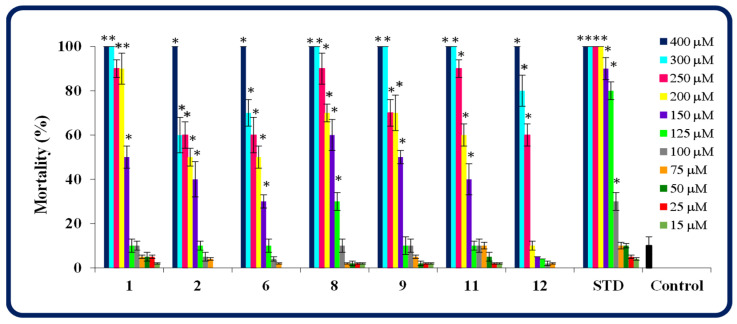
The 96-hpf mortality of zebrafish. STD—a standard drug pemetrexed. Data represent the mean ± SD of three independent experiments under similar conditions. *—statistically different from the control group (*p* < 0.05, Student’s *t*-test).

As shown in Figure 7, the mortality of zebrafish was dependent on the concentration of the tested compound/pemetrexed. Compared to the control group, the triazinone **12** at concentrations up to 200 µM, molecules **1**, **2**, **6**, **9**, **11** at concentrations up to 125 µM, the structure **8** at concentrations up to 100 µM did not affect the survival rates in zebrafish. It is worth noting that compounds **2**, **6**, and **12** caused complete lethality only at the highest concentration tested (400 µM), while compounds **1**, **8**, **9**, and **11** also at 300 µM. In turn, pemetrexed did not affect zebrafish survival only at concentrations up to 75 µM, while it caused 100% mortality at a concentration of 200 µM.

Taking into account the 96-h mortality of zebrafish, it was established that the maximal non-lethal concentrations for the investigated compounds ranged from 100 to 183 µM (Table 3). The MNLC was the highest for the triazinone **12** (containing the 2-methyl-substituted phenyl and 2-chloro-substituted benzyl), and the lowest for the parent compound **1** (with the unsubstituted phenyl. In turn, the half maximal lethal concentrations were found to be in the range of 144 µM (the structure **1**) to 249 µM (the compound **12**). Interestingly, the standard drug proved to be more toxic (MNLC = 75 µM and LC_50_ = 101 µM) than all disubstituted heterofused 1,2,4-triazinones tested, indicating that these molecules are safer for zebrafish than pemetrexed (Table 3).

As our research showed, the introduction of a benzyl or 2-chlorobenzyl substituent instead of an isopropyl group at *C*3 of the 1,2,4-triazinone system had a beneficial effect, because it significantly reduced the toxicity to zebrafish of the title class of molecules. This was particularly noticeable in the case of heterofused triazinones **1**, **2** (bearing an unsubstituted phenyl as R^1^) and **6** (containing a 4-methylphenyl as R^1^). For these compounds both the MNLC (for **1**: 100 µM, for **2** and **6**: 125 µM) and LC_50_ (for **1**: 144 µM, for **2**: 233 µM, for **6**: 236 µM) values were much higher than the values previously determined for their isopropylated congeners, i.e., for structures with the unsubstituted (MNLC = 63.3 µM, LC_50_ = 117 µM) and 4-methyl-substituted (MNLC = 50 µM, LC_50_ = 105 µM) phenyl ring [27].

**Figure 8:**
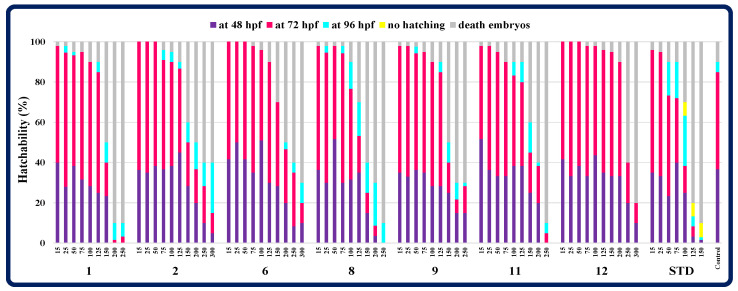
The ability to hatch of zebrafish embryos in the control and compound/standard drug-treated groups. STD—a standard drug pemetrexed.

When examining the effect of triazinones on the ability to hatch of *Danio rerio*, all viable embryos from compound-treated groups left the chorion before 96 hpf, in contrast to zebrafish exposed to pemetrexed at concentrations higher than 75 µM, in which hatching was inhibited (Figure 8). Therefore, all the molecules tested had no negative impact on the hatchability of embryos.

**Figure 9:**
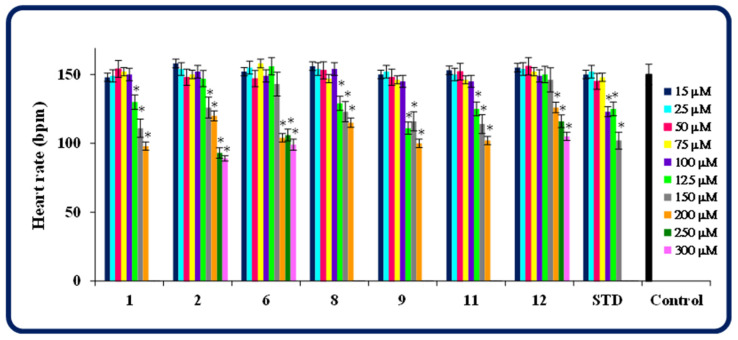
Heart rate in beats per minute (bpm) in zebrafish larvae exposed to test compounds/pemetrexed. STD—a standard drug pemetrexed. Data represent the mean ± SD of three independent experiments under similar conditions. *—statistically different from the control group (*p* < 0.05, Student’s *t*-test).

Another parameter assessed in this study was the cardiac function in compound/standard drug-treated larvae. Therefore, in each group, the heart rate per minute was calculated. The conducted research showed that molecules **6**, **12** at concentrations up to 150 µM, **2** at concentrations up to 125 µM, and **1**, **8**, **9**, **11** at concentrations up to 100 µM had no significant effect on the heart rate in zebrafish, compared to the control. In turn, in the groups exposed to higher concentrations of heterofused triazinones or pemetrexed at concentrations above 75 µM, a reduced number of heartbeats was observed (Figure 9). This indicates that all the compounds tested are less cardiotoxic to *Danio rerio* than pemetrexed.

**Figure 10:**
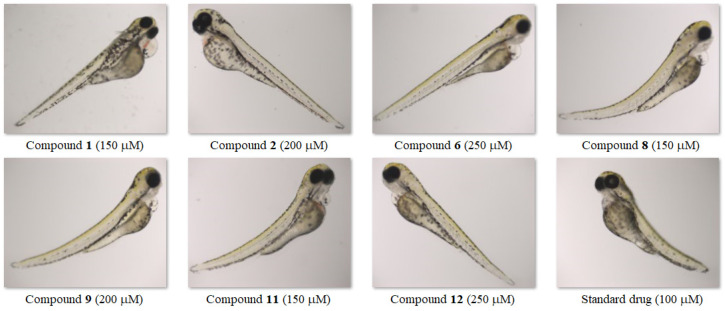
Morphological abnormalities in zebrafish larvae induced by 96-h exposure to the tested compounds/pemetrexed.

In order to assess the acute toxicity of the tested molecules, the occurrence of developmental malformations in zebrafish was also analysed. Most larvae treated with heterofused triazinones developed normally. Only exposure to the highest concentrations of the tested compounds resulted in various developmental abnormalities, such as the abnormal body shape and pericardial or yolk sac oedema. However, these malformations were observed less frequently than in larvae exposed to the standard drug. Figure 10 presents the most severe developmental abnormalities in zebrafish larvae induced by 96-h exposure to the tested compounds/pemetrexed.

**Figure 11:**
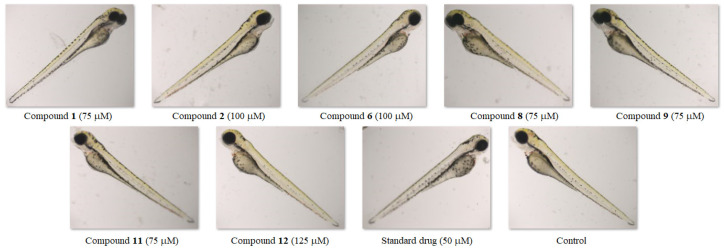
Zebrafish larvae exposed to the highest concentration of compound/standard drug that did not induce phenotypic abnormalities.

Taking into account all observed lethal and sub-lethal endpoints, the NOAEC was established at 125 µM for the triazinone **12**, 100 µM—for structures **2** and **6**, and 75 µM—for compounds **1**, **8**, **9**, and **11**, while the LOAEC was 150, 125, and 100 µM, respectively. It is noteworthy that the NOAEC and LOAEC values for the standard drug were lower than those for all the tested molecules and amounted to 50 and 75 µM, respectively (Table 3). This proves that disubstituted heterofused triazinones are safer for the developing zebrafish than pemetrexed. Additionally, the no observed adverse effect concentrations for compounds **1**, **2**, and **6** from this class of heterocycles were found to be higher (75 µM, 100 µM, and 100 µM, respectively) than those determined—in an earlier investigation—for their isopropylated congeners containing the unsubstituted or 4-methyl-substituted phenyl ring, for which the NOAEC was 50 µM [27]. This demonstrates that the presence of unsubstituted/substituted benzyl at *C*3 of the as-triazinone template is advantageous in terms of toxicity to zebrafish. Figure 11 shows representative 96-h-old zebrafish larvae exposed to the highest concentration of compound/standard drug at which no adverse effects were observed.

### 2.4. The Effect of the Investigated Compounds (***1**–**12***) on Red Blood Cells

**Figure 12:**
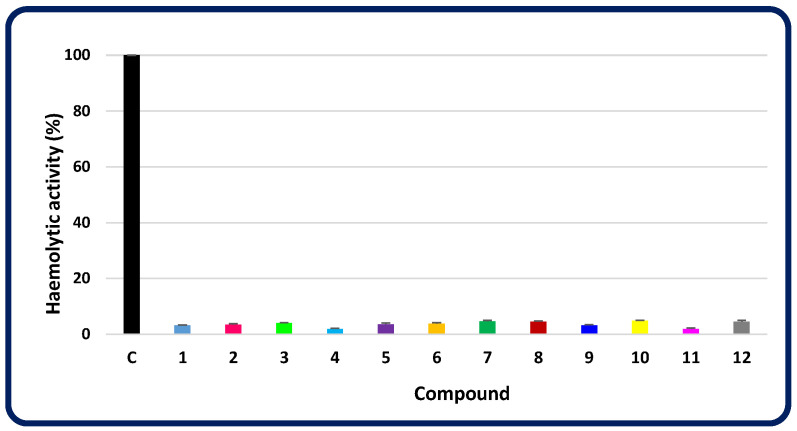
Haemolytic activities of the investigated compounds (**1**–**12**) in relation to the control (C—triton X-100).

A good model for testing the cytotoxicity of drug candidates are the most numerous blood cells, i.e., erythrocytes [46,47]. Therefore, the ability of disubstituted heterofused 1,2,4-triazinones (**1**–**12**) to induce haemolysis was investigated ex vivo in a mammalian red blood cell model. It turned out that none of these compounds had a significant haemolytic effect, as the haemolytic activity of each of them was less than 5% (Figure 12). At the same time, triton X-100, which was used as a positive control, caused 100% haemolysis. This confirms the good haemocompatibility of all the molecules, which is of particular importance due to the potential therapeutic utility of these drug candidates.

**Figure 13:**
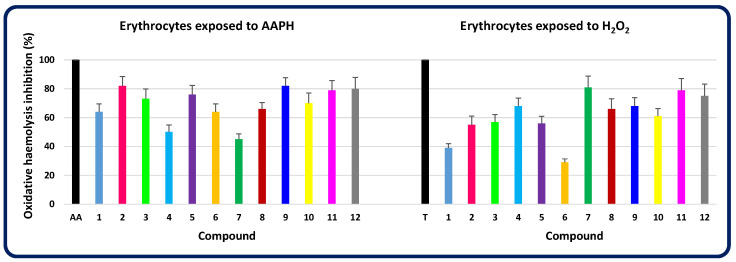
Percentage of inhibition of oxidative haemolysis by the investigated compounds (**1**–**12**) in relation to antioxidant standards. AAPH—2,2′-azobis(2-methylpropionamidine)dihydrochloride; H_2_O_2_—hydrogen peroxide; AA—ascorbic acid; T—trolox (6-hydroxy-2,5,7,8-tetramethylchroman-2-carboxylic acid).

In turn, the ability of compounds **1**–**12** to counteract oxidative stress-induced haemolysis was assessed ex vivo in a model of mammalian red blood cells exposed to reactive oxygen species, such as AAPH-derived peroxyl radicals or hydrogen peroxides. These oxidants, through protein oxidation and lipid peroxidation, damage the erythrocyte membrane and thus cause haemolysis. Among disubstituted heterofused 1,2,4-triazinones, molecules **2**, **5**, **7**, **9**, **11**, and **12** were found to be the most active in protecting erythrocytes against oxidative damage (Figure 13). Molecules **2**, **5**, **9**, **11**, and **12** turned out to be the most effective in counteracting haemolysis induced by peroxyl radicals, as their antihaemolytic activity was 82, 76, 82, 79, and 80% of the activity of ascorbic acid (a positive control), respectively. In turn, the strongest inhibition of hydrogen peroxide-induced haemolysis was observed after incubation of erythrocytes with compounds **7**, **11**, and **12**. Their activity was 81, 79, and 75%, respectively, of that of trolox (a positive control). Therefore, molecules **2**, **5**, **7**, **9**, **11**, and **12**—which most protect red blood cells against oxidative damage—may be helpful in the development of preventive agents against free radical-mediated diseases.

These results confirm that all tested disubstituted heterofused 1,2,4-triazinones are safe for erythrocytes. The lack of haemolytic activity and effective protection of red blood cells against oxidative damage are favourable properties of these drug candidates.

## 3. Materials and Methods

### 3.1. Disubstituted Heterofused Triazinones (***1**–**12***)

Imidazolidinoannelated 1,2,4-triazinones, which are substituted at *C*3 and *N*8, i.e., 3-[(R^2^-phenyl)methyl]-8-(R^1^-phenyl)-7,8-dihydroimidazo[2,1-*c*][1,2,4]triazin-4(6*H*)-ones (**1**–**12**), were synthesised as previously published [1]. Pure samples of all the compounds were obtained after recrystallisation of the crude reaction products. The structures of all the molecules subjected to thermal investigations have been verified by their spectroscopic data (i.e., ^1^H NMR spectra—which confirmed the attendance of all protons present within each molecule, IR spectra with the most characteristic absorption band of the ring carbonyl group in the range of 1675–1695 cm^−1^), and microanalyses being within ± 0.4% of the theoretical values [1]. In addition, the purity and homogeneity of all the samples (subjected to thermal studies) have been corroborated in our chromatographic investigations [3,4]. Each compound sample was stored at room temperature in a brown glass bottle.

### 3.2. Thermal Analysis Methods

Thermal analyses (TG and DSC methods) were conducted in a flowing air atmosphere (v = 0.75 dm^3^ h^−1^) using the SETSYS 16/18 analyser (Setaram, Caluire-et-Cuire, France). Samples (5–7 mg) placed in 100 µL alumina crucibles were heated in the temperature range of 30–1000 °C at a heating rate of 10 °C min^−1^. The indium standard was used for temperature and heat flow calibration. Coupled TG-FTIR measurements were carried out using the Q5000 (TA Instruments, New Castle, DE, USA) apparatus, connected to the Nicolet 6700 (Thermo Scientific, Waltham, MA, USA) spectrophotometer, in a flowing nitrogen atmosphere (25 cm^3^ min^−1^). Approximately 30 mg samples were placed in platinum crucibles and heated from room temperature to 700 °C at a heating rate of 20 °C min^−1^.

### 3.3. Toxicity Studies on a Zebrafish Model

The FET (Fish Embryo Acute Toxicity) test was performed in the zebrafish (*Danio rerio*) model according to the OECD Guidelines for the Testing of Chemicals [38], in compliance with European legislation on the protection of animals used for scientific purposes [48]. Due to the fact that zebrafish embryos/larvae up to 120 h old are not protected animal stages, approval from the ethics committee was not necessary to conduct this study [48]. All experimental procedures on zebrafish were carried out at the Experimental Medicine Centre of the Medical University of Lublin, Poland.

Zebrafish eggs were obtained by random mating of wild-type adult males and females (2:1), which were maintained in a water recirculating system at controlled temperature (28 ± 0.5 °C) and photoperiod (14 h light/10 h dark). After fertilisation, eggs were placed in Petri dishes (Costar, Corning Inc., Glendale, AZ, USA) (100 eggs/dish) containing embryonic medium (E3 medium: a purified water with 17.4 µM NaCl, 0.21 µM KCl, 0.18 µM Ca(NO_3_)_2_, and 0.12 µM MgSO_4_, pH = 7.1–7.3). Unfertilised, coagulated, and damaged eggs were removed after observation under a SteREO Discovery.V8 optical microscope with a camera (Carl Zeiss Microscopy GmbH, Göttingen, Germany). The remaining eggs were distributed into sterile 24-well plates (Costar, Corning Inc., Glendale, AZ, USA) (one embryo per well) filled with 2 mL of test solution (i.e., a solution prepared by dissolving the compound or pemetrexed (Sigma-Aldrich, St Louis, MO, USA) in DMSO (Sigma-Aldrich, St Louis, MO, USA) and then diluting in E3 medium to obtain concentrations in the range of 15 to 400 µM). To ensure that the real concentrations of the test solutions did not fall under 80% of their nominal values, they were replaced every day. The treated groups consisted of 20 embryos exposed to the compound/standard drug at one of 11 tested concentrations (15, 25, 50, 75, 100, 125, 150, 200, 250, 300 or 400 µM), while the control group consisted of 20 embryos placed in E3 medium only. Since the concentration of the solvent (DMSO) in our study had no effect on the development of zebrafish, a DMSO control group was not required. The covered plates were stored in an incubator (IN 110 Memmert GmbH, Schwabach, Germany) at controlled temperature (28 ± 0.5 °C) and photoperiod (12 h light/12 h dark) for 96 h. Every 24 h, mortality, hatching rate, heart rate, and the frequency and type of developmental defects were observed under a microscope. After completing the research, the larvae were euthanised by an overdose of tricaine (300 mg L^−1^ solution; Sigma-Aldrich, St Louis, MO, USA). All experiments were performed in triplicate under similar conditions.

### 3.4. Investigation of the Effect of Compounds ***1**–**12*** on Erythrocytes

The influence of disubstituted heterofused 1,2,4-triazinones on erythrocytes was investigated in the haemolytic assay as well as in the oxidative haemolysis inhibition assay, according to the procedures described earlier [5,47]. Blood for both tests was collected from rats (male Wistar rats; 7 weeks old; 240 g) kept at the Experimental Medicine Centre of the Medical University of Lublin, Poland.

## 4. Conclusions

The study presents, for the first time, the thermal characterisation and toxicity profile of potential drugs from a class of disubstituted heterofused triazinones. These compounds are thermally stable in both air and nitrogen atmospheres, and their decomposition occurs above their melting temperatures, which range from 162 °C to 264 °C. Further heating leads to a two-step (air) or a one-step (nitrogen) degradation. The type of R^1^ and R^2^ substituents at the phenyl and benzyl moiety, respectively, has a massive influence on both the thermostability of the compounds and the degradation mechanisms. Compounds **3** and **7** exhibit the highest melting point as well as the highest thermal stability in both atmospheres, which may be correlated with their structures, where the chloro group is located at the *para* position of the benzyl moiety. This arrangement appears to be energetically favoured and provides the structure with the highest thermal stability. The spectra of gaseous products formed during the degradation of compounds **1**–**12** are very complex. Two groups of volatile decomposition products can be distinguished: these associated with the degradation of the bicyclic system, which are formed first, and those containing aromatic groups. The chemical nature of the identified benzene derivatives is closely related to the type of R^1^ and R^2^ substituents at the phenyl and benzyl moiety, respectively.

The results of biological studies revealed that the investigated heterofused triazinones are safer for the early-life stages of zebrafish than a clinically approved anticancer drug and confirmed that the presence in their molecules of unsubstituted/substituted benzyl moiety is advantageous in terms of toxicity. The MNLC, LC_50_, NOAEC, and LOAEC values for these compounds were higher than those for pemetrexed, and exposure to them caused significantly weaker and less frequent developmental abnormalities in *Danio rerio* than exposure to this drug. At the same time, all the tested molecules were not toxic to red blood cells, because they did not cause haemolysis. Moreover, some of them—**2**, **5**, **7**, **9**, **11**, and **12**—protected erythrocytes against oxidative damage by preventing haemolysis induced by reactive oxygen species. Based on their beneficial toxicity profile and antihaemolytic activity, this class of compounds may be intended for more detailed in vivo studies.

Disubstituted heterofused triazinones appear to be suitable molecules for further development in the drug discovery process due to their favourable thermal properties and safety/toxicity profile.

## Figures and Tables

**Table 1:** Thermal data for the melting process of disubstituted heterofused triazinones (**1**–**12**) evaluated by DSC.

Compound	R^1^	R^2^	Melting Process		
			T_onset_ [°C]	T_peak_ [°C]	ΔH_m_ [kJ·mol^−1^]
**1**	H	H	216	219	33.19
**2**	H	2-Cl	238	242	41.52
**3**	H	4-Cl	261	264	51.62
**4**	4-CH_3_	H	220	227	21.08
**5**	4-CH_3_	3-CH_3_	205	209	34.60
**6**	4-CH_3_	2-Cl	236	240	32.11
**7**	4-CH_3_	4-Cl	250	254	46.48
**8**	4-OCH_2_CH_3_	H	182	187	33.69
**9**	4-OCH_2_CH_3_	2-Cl	208	211	52.82
**10**	4-OCH_2_CH_3_	3-Cl	158	162	28.11
**11**	4-OCH_2_CH_3_	4-Cl	219	221	35.79
**12**	2-CH_3_	2-Cl	159	164	34.03

T_onset_—the onset temperature of endothermic effect; T_peak_—the melting peak temperature; ΔH_m_—the melting enthalpy.

**Table 2:** Thermal data for the decomposition process of disubstituted heterofused triazinones (**1**–**12**).

Compound	R^1^	R^2^	Decomposition Process		
			Step 1		Step 2
			ΔT_1_ [°C]	Δm_1_ [%]	ΔT_2_ [°C]
**1**	H	H	264–468	54.54	468–690
**2**	H	2-Cl	275–472	53.68	472–669
**3**	H	4-Cl	276–453	45.93	453–661
**4**	4-CH_3_	H	255–489	50.22	489–676
**5**	4-CH_3_	3-CH_3_	260–415	47.13	468–674
**6**	4-CH_3_	2-Cl	264–454	49.94	454–641
**7**	4-CH_3_	4-Cl	271–465	44.21	465–674
**8**	4-OCH_2_CH_3_	H	242–454	51.02	454–674
**9**	4-OCH_2_CH_3_	2-Cl	243–453	47.91	453–668
**10**	4-OCH_2_CH_3_	3-Cl	214–472	50.35	472–690
**11**	4-OCH_2_CH_3_	4-Cl	251–468	45.36	468–686
**12**	2-CH_3_	2-Cl	258–472	57.96	472–664

ΔT_1_, ΔT_2_—temperature ranges of decomposition stages; Δm_1_—mass loss.

**Table 3:** The MNLC, LC_50_, NOAEC, and LOAEC values of the selected compounds (**1**, **2**, **6**, **8**, **9**, **11**, **12**) and a standard drug for the developing zebrafish.

Compound	R^1^	R^2^	MNLC (µM) ^a^	LC_50_ (95% CL ^b^, µM) ^c^	NOAEC (µM)	LOAEC (µM)
**1**	H	H	100 ± 0.0	144 (118–176)	75	100
**2**	H	2-Cl	125 ± 0.0	233 (192–282)	100	125
**6**	4-CH_3_	2-Cl	125 ± 0.0	236 (200–279)	100	125
**8**	4-OCH_2_CH_3_	H	108 ± 14.4	169 (142–202)	75	100
**9**	4-OCH_2_CH_3_	2-Cl	108 ± 14.4	177 (148–213)	75	100
**11**	4-OCH_2_CH_3_	4-Cl	117 ± 14.4	186 (158–219)	75	100
**12**	2-CH_3_	2-Cl	183 ± 28.9	249 (216–286)	125	150
Standard drug ^d^			75 ± 0.0	101 (80–127)	50	75

^a^—The mean ± SD (from three independent experiments), ^b^ CL—confidence limit, ^c^—calculated using the probit analysis [45], ^d^ pemetrexed. MNLC—the maximum concentration of the compound/standard drug that does not result in a statistically different mortality of zebrafish embryos/larvae compared to the control group during the experimental period; LC_50_—the lowest concentration of the compound/standard drug causing 50% mortality of zebrafish embryos/larvae during the experiment; NOAEC—the highest concentration of the compound/standard drug at which no adverse effects are observed; LOAEC—the lowest concentration of the compound/standard drug at which adverse effects are observed.

## Data Availability

Data are contained within the article.
